# Supplementary material for: Identification of photoperiod-induced specific miRNAs in the adrenal glands of Sunite sheep (Ovis aries)
Source: Front Vet Sci. 2022 Jul 22;9:888207. doi: 10.3389/fvets.2022.888207 (PMC9354845; doi:10.3389/fvets.2022.888207)
Supplement: Supplementary file 1 [file Data_Sheet_1.ZIP › Supplementary file/miRDeep2 file.zip!/miRDeep2 file/Know_Structure/SP42A1/oar-mir-370.pdf]

miRBase precursor : oar-mir-370
Total read count : 6310
oar-miR-370-5p read count : 25
oar-miR-370-3p read count : 6280
remaining reads : 5

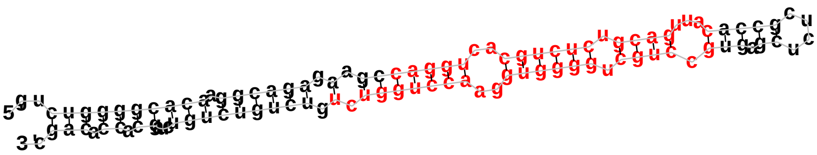

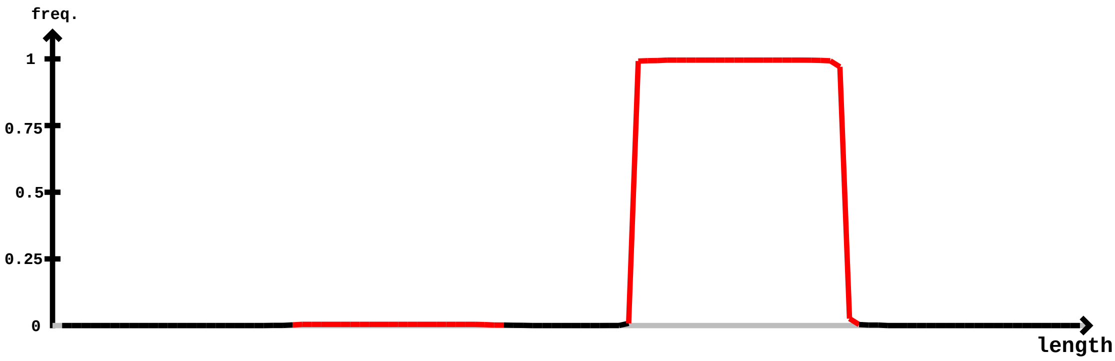


```
                                                    oar-miR-370-3p
                                oar-miR-370-5p
5'- gucuggggcacaaggcagagaagccaggucacgucucugcaguuacaccgcucucgagugccugcugggguggaaccuggucugucugucugucuagcaccacagc   -3'   exp
    ..((((((((((.((((((.(.(((((((..((((((.((((...(((((....)).))).)))).))))))..))))))).).)))))))))...)).)).))).   reads   mm   sample
    .....................agccaggucacgucucugcagu...............................................................   4       0    seq
    .......................ccaggucacgucucugcagu...............................................................   2       0    seq
    .......................ccaggucacgucucugcaguu..............................................................   9       0    seq
    .......................ccaggucacgucucugcaguua.............................................................   1       0    seq
    ........................caggucacgucucugcaguu..............................................................   1       0    seq
    ........................caggucacgucucugcaguuac............................................................   5       0    seq
    ........................caggucacgucucugcaguuaca...........................................................   2       0    seq
    ........................caggucacgucucugcaguuacac..........................................................   5       0    seq
    ........................................................agugccugcugggguggaaccuggu.........................   1       0    seq
    ..........................................................ugccugcugggguggaaccu............................   1       0    seq
    ..........................................................ugccugcugggguggaaccug...........................   1       0    seq
    ..........................................................ugccugcugggguggaaccugg..........................   5       0    seq
    ..........................................................ugccugcugggguggaaccuggu.........................   44      0    seq
    ..........................................................ugccugcugggguggaaccuggucu.......................   1       0    seq
    ...........................................................gccugcugggguggaacc.............................   2       0    seq
    ...........................................................gccugcugggguggaaccu............................   4       0    seq
    ...........................................................gccugcugggguggaaccug...........................   9       0    seq
    ...........................................................gccugcugggguggaaccugg..........................   137     0    seq
    ...........................................................gccugcugggguggaaccuggu.........................   5906    0    seq
    ...........................................................gccugcugggguggaaccugguc........................   138     0    seq
    ...........................................................gccugcugggguggaaccuggucu.......................   9       0    seq
    ............................................................ccugcugggguggaaccuggu.........................   5       0    seq
    ............................................................ccugcugggguggaaccugguc........................   2       0    seq
    ............................................................ccugcugggguggaaccuggucu.......................   2       0    seq
    .............................................................cugcugggguggaaccuggu.........................   1       0    seq
    .............................................................cugcugggguggaaccugguc........................   1       0    seq
    .............................................................cugcugggguggaaccuggucugu.....................   1       0    seq
    ..............................................................ugcugggguggaaccuggucugu.....................   11      0    seq
```
